# Factors influencing the progression from prehypertension to hypertension among Chinese middle-aged and older adults: a 2-year longitudinal study

**DOI:** 10.1186/s12889-022-14410-3

**Published:** 2023-02-15

**Authors:** Zhen Li, Lianmeng Cao, Ziyu Zhou, Maozhi Han, Chang Fu

**Affiliations:** 1 Yantai Center for Disease Control and Prevention, No.17 Fuhou Road, Yantai, 264003 Shandong China; 2 Department of Gastrointestinal Surgery Bariatric and Metabolic Surgery, Binzhou Medical University Hospital, No. 661 2nd Huanghe Road, Binzhou, 256603 Shandong China; 3 Department of Anesthesiology, the 80Th Army Hospital, No. 256 Beigongxijie Rd, Weifang, Shandong, 261021 China; 4 Department of Pharmacy, the 80Th Army Hospital, No. 256 Beigongxijie Rd. , Weifang, 261021 Shandong China; 5 Department of Health Service and Management,School of Public Health and Management, Binzhou Medical University, No.346 Guanhai Road, Yantai, 264003 Shandong China

**Keywords:** Prehypertension, Hypertension, Chinese middle aged and older adults, Factors influencing hypertension, Gender difference

## Abstract

**Background:**

This study aimed to investigate the proportion of prehypertension cases progressing to hypertension among Chinese middle-aged and elderly populations over a 2-year period and related influencing factors.

**Methods:**

Data were obtained from the China Health and Retirement Longitudinal Study, and 2,845 individuals who were ≥ 45 years old and prehypertensive at baseline were followed from 2013–2015. Structured questionnaires were administered, and blood pressure (BP) and anthropometric measurements were performed by trained personnel. Multiple logistic regression analysis was done to investigate factors associated with prehypertension progressing to hypertension.

**Results:**

Over the 2-year follow-up, 28.5% experienced progression of prehypertension to hypertension; this occurred more frequently in men than women (29.7% vs. 27.1%). Among men, older age (55–64 years: adjusted odds ratio [aOR] = 1.414, 95% confidence interval [CI]:1.032–1.938; 65–74 years: aOR = 1.633, 95%CI: 1.132–2.355; ≥ 75 years: aOR = 2.974, 95%CI: 1.748–5.060), obesity (aOR = 1.634, 95%CI: 1.022–2.611), and number of chronic diseases (1: aOR = 1.366, 95%CI: 1.004–1.859; ≥ 2: aOR = 1.568, 95%CI: 1.134–2.169) were risk factors for progression to hypertension whereas being married/cohabiting (aOR = 0.642, 95% CI: 0.418–0.985) was a protective factor. Among women, risk factors included older age (55–64 years: aOR = 1.755, 95%CI: 1.256–2.450; 65–74 years: aOR = 2.430, 95%CI: 1.605–3.678; ≥ 75 years: aOR = 2.037, 95% CI: 1.038–3.995), married/cohabiting (aOR = 1.662, 95%CI: 1.052–2.626), obesity (aOR = 1.874, 95%CI: 1.229–2.857), and longer naps (≥ 30 and < 60 min: aOR = 1.682, 95%CI: 1.072–2.637; ≥ 60 min: aOR = 1.387, 95%CI: 1.019–1.889).

**Conclusions:**

Chinese middle-aged and elderly individuals experienced a risk of prehypertension progressing to hypertension over a 2-year period, although the influencing factors differed by sex; this should be considered in interventions.

## Introduction

Treating hypertension is an important public health issue, because more than one-fourth of the adult population worldwide is afflicted with the disease [1]. Hypertension can cause damage to the brain, heart, kidneys, blood vessels, and other important organs, leading to clinical complications [2–4] and the prolonged course of the disease places a serious burden on individuals [5].

Those with prehypertension exist in a special group between those with normal blood pressure (BP) and those with high BP. The Seventh Report of the Joint National Committee on Prevention, Detection, Evaluation, and Treatment of High Blood Pressure was the first to propose the concept of prehypertension [6]. Prehypertension progresses to hypertension, which is related to high morbidity and mortality rate due to the risk of cardiovascular disorders and stroke [7]. In addition to affecting a large proportion of the population, the condition increases the risk of developing hypertension and experiencing cardiovascular events in the future. Two-thirds of untreated prehypertension cases can progress to hypertension within 4 years [8], and a previous study reported that those with prehypertension at baseline had a 45% higher risk of chronic noncommunicable disease events than those with normal baseline BP [9, 10].

The prevalence of prehypertension among adults has reached about 40% worldwide [11]. In 1999–2000 and 2011–2012, the prevalence of prehypertension was 31.2% and 28.2%, respectively, in adults in the United States [12]. In Chinese adults, the prevalence ranged from 36.4%-41.3% from 2007 to 2015 [13, 14]. Many studies have shown that in those with prehypertension, a precursor of hypertension, the risk of cardiovascular events is significantly higher than that in those with normal BP [15, 16]. One study indicated that if the BPs of those with prehypertension could have been reduced to the optimal range (< 120/80 mmHg), 25% of the hypertension cases among men and 21% among women would have been prevented [17]. Some studies have also shown that effective interventions for prehypertension can reduce the risk of cardiovascular disease [6, 18]. Although some studies have focused on the conversion of prehypertension in the early stage of the disease, their research had some limitations [17, 19, 20]. First, the studies mostly focused on predicting the risk of hypertension. Second, the sample sizes were not large enough to investigate the natural history of prehypertension in the general population. Third, few studies have considered the potential sex differences when investigating the progression from prehypertension to hypertension. In recent years, considerable attention has been given to gender differences in epidemiology, pathophysiology, and treatment of human diseases. The focus of much of the literature addressing gender differences in cardiovascular disease has been on the unique presentation of the disease in women and differences in natural history [21]. Previous studies have emphasized the fact that hypertension is more common in older women compared with men [22, 23]. In general, men and women differ in terms of physiology, social roles, and living habits; therefore, the factors influencing the progression of prehypertension to hypertension may also differ.

In China, with the increasing aging of the population, the prevalence of hypertension has become one of the highest of all noncommunicable chronic diseases. According to a report based on the Chinese National Nutrition and Health Survey, the prevalence of hypertension was 45.8% among males and 33.0% among females in 2011 [24]. However, few studies have investigated the factors influencing the progression from prehypertension to hypertension among Chinese middle-aged and elderly individuals in the early stage of the disease. The aim of this study was to investigate the proportion of prehypertension cases that progress to hypertension and to identify the factors influencing this progression, taking into consideration potential sex differences.

## Methods

### Data source

**Fig.1:**
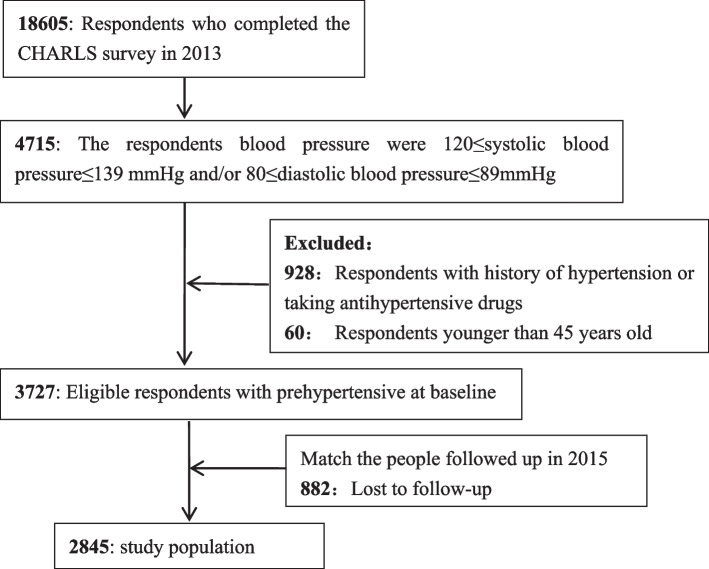
Flow chart of the study selection process

The China Health and Retirement Longitudinal Study (CHARLS) was conducted by the National School of Development of Peking University. A multistage stratified design was used in this survey, and the survey covered 150 counties/districts and 450 villages/resident committees from 28 provinces in China. The 2013 survey involved 18,605 respondents and the data from the follow-up assessment conducted in 2015 and involved 21,096 respondents. In the present study, baseline data were collected from middle-aged and elderly individuals who were over 45 years old and with prehypertension in 2013, and the follow-up survey in 2015 was used to study the progression and factors influencing the progression from prehypertension to hypertension. Finally, a total of 2,845 subjects were included in this study (Fig. 1).

### Measurement and definitions

The face-to-face survey questionnaire and related clinic examination were conducted by trained investigators. Before the BP measurement, the subjects were abstained from physical activity, smoking, and drinking alcohol or coffee. The same type of OMRON HEM-7200 sphygmomanometer was used for all BP measurements. The BP of each individual was measured three times, and the average was taken as the final BP value for each individual. Normal BP was defined as systolic BP < 120 mmHg and diastolic BP < 80 mmHg and without the use of antihypertensive medication [6]. Prehypertension was defined as 120 mmHg ≤ systolic BP ≤ 139 mmHg and/or 80 mmHg ≤ diastolic BP ≤ 89 mmHg [6]. Individuals with a history of hypertension or those taking antihypertensive drugs were excluded. Hypertensive individuals were defined as those who reported they were told by a doctor that they had high BP and/or those with a systolic BP ≥ 140 mmHg or a diastolic BP ≥ 90 mmHg or those using antihypertensive medications [6]. The survey methods and quality control standards were kept consistent with the measurements taken in 2013 and 2015.

### Covariates

Trained personnel administered questionnaires to collect self-reported information on sociodemographic variables, health-related behaviors, and prior diagnosed diseases. Participants were divided into the following age brackets: 45–54, 55–64, 65–74, and ≥ 75. A region was classified as either an urban or a rural area. Education levels were categorized as primary school or below, junior/secondary/vocational school, college and higher. Marital status was classified as being either married/cohabiting or single (including divorced, separated, widowed, or having never been married). Body mass index (BMI) was calculated as weight (kg)/height(m)^2^ and categorized as underweight (< 18.5 kg/m^2^), normal weight (18.5–23.9 kg/m^2^), overweight (24–27.9 kg/m^2^) or obese (≥ 28 kg/m^2^)[25]. The standard waist circumference indicating abdominal obesity was ≥ 85 cm for men and ≥ 80 cm for women. Smoking status and alcohol habits were classified as ‘yes’ or ‘no.’ Sleep duration was categorized into the following five groups: < 6, ≥ 6 and < 7, ≥ 7 and < 8, ≥ 8 and < 9, and ≥ 9 h/night [26]. Nap times were classified into the following five groups: 0: > 0 and < 30; ≥ 30 and < 60; and ≥ 60 min/day[5]. Self-rated assessments of health status were categorized into the following three groups: good, fair, or poor. A number of chronic diseases (such as dyslipidemia, diabetes, hyperglycemia, chronic kidney disease, and others, except for hypertension) were reported by participants. Social activities were defined as one’s involvement in activities[27] (such as interacting with friends, volunteering, and participating in hobby groups, sports groups, and community-related organizations).

### Statistical analysis

Statistical Package for the Social Sciences (SPSS) Statistics Version 21.0 (IBM Corp., Armonk, NY, USA) software was used for the statistical analysis. Continuous variables are presented as the mean values and standard deviation (SD). Two tailed *t*-tests were used to compare continuous variables between two groups(*t*-value), whereas one-way ANOVAs were used to compare the three groups (*F*-value). Categorical variables are described using percentages and compared using chi-square tests (*χ2*). Wilcoxon rank sum testing was applied to ordinal categorical variables (*Z*-values), whereas Kruskal–Wallis testing was applied to multiple independent samples of one-way ordered variables(*H*-value). Multiple logistic regression analyses were used to identify the factors influencing the progression from prehypertension to hypertension. Adjusted odds ratios (aORs) are presented with 95% confidence intervals (CIs), and *P* < 0.05 was considered statistically significant.

### Ethical approval

CHARLS has been approved by the Ethical Review Committee of Peking University. Ethical approval of the present data analysis was not needed. All subjects gave their informed consent for inclusion before participating in the survey. All methods in the present study were carried out in accordance with relevant guidelines and regulations.

## Results

### Baseline characteristics of study respondents with prehypertension

This study followed 2,845 individuals (1,481 men and 1,364 women). The mean age of the study respondents was 59.65 ± 9.20 years. The mean BPs (systolic and diastolic) were 128.29 ± 5.93 and 76.16 ± 7.23 mmHg, respectively, in men and 128.42 ± 6.08 and 75.38 ± 7.31 mmHg, respectively, in women. Abdominal obesity was more common in women than in men (78.4% vs. 52.5%). Most participants lived in rural areas (86.6%). The majority of men and women were married/cohabiting (91.0% vs. 85.2%, respectively). The education level predominantly consisted of those with primary school education and lower (67.3%). Only 18.9% of participants slept 7–8 h at night, and 43.3% of participants did not nap habitually. Daytime napping was more commonly practiced in men than in women (63.8% vs. 51.0%). In terms of social activities, 56.7% of participants had participated in them in the last month. More than half of the men smoked (54.6%), whereas few women (19.3%) did. Similarly, 71% of men drank alcohol habitually, compared with only 20.8% of women. A normal BMI was observed in 50.8% of participants. Approximately 32.3% of the participants had more than two chronic diseases. There was a significant difference in diastolic BP, abdominal obesity, marital status, education level, alcohol habits, current smoking status, BMI, sleep duration, nap length, health status, and number of chronic diseases among men and women (*P* < 0.05, Table 1).

**Table 1:** Demographic and clinical characteristics of prehypertension

Characteristic	Total(*n* = 2845)	Men(*n* = 1481)	Women(*n* = 1364)	*χ*^*2*^*/Z/ t*	*P-*value
SBP(mmHg)	128.35 ± 6.00	128.29 ± 5.93	128.42 ± 6.08	-0.590	0.555^a^
DBP(mmHg)	75.79 ± 7.28	76.16 ± 7.23	75.38 ± 7.31	2.858	0.004^a^
Age group(years), n(%)				6.568	0.087^b^
45–54	925(32.5)	454(30.7)	471(34.5)		
55–64	1134(39.9)	594(40.1)	540(39.6)		
65–74	567(19.9)	316(21.3)	251(18.4)		
≥ 75	217(7.6)	116(7.8)	101(7.4)		
Missing, n(%)	2(0.1)	1(0.1)	1(0.1)		
Marital status, n(%)				22.629	< 0.001^c^
Single	336(11.8)	134(9.0)	202(14.8)		
Married or cohabiting	2509(88.2)	1347(91.0)	1162(85.2)		
Region, n(%)				0.396	0.529^c^
Urban	370(13.0)	187(12.6)	183(13.4)		
Rural	2463(86.6)	1288(87.0)	1175(86.1)		
Missing, n(%)	12(0.4)	6(0.4)	6(0.4)		
Education level, n(%)				128.042	< 0.001^b^
Primary and lower	1916(67.3)	856(57.8)	1060(77.7)		
Junior/secondary/vocational school	840(29.5)	565(38.1)	275(20.2)		
College and higher	89(3.1)	60(4.1)	29(2.1)		
Current smoking status, n(%)				377.723	< 0.001^c^
Yes	1072(37.7)	809(54.6)	263(19.3)		
No	1773(62.3)	672(45.4)	1101(80.7)		
Alcohol habits, n(%)				719.482	< 0.001^c^
Yes	1336(47.0)	1052(71.0)	284(20.8)		
No	1505(52.9)	427(28.8)	1078(79.0)		
Missing, n(%)	4(0.1)	2(0.1)	2(0.1)		
Social activity, n(%)				1.435	0.231^c^
No	1231(43.3)	625(42.2)	606(44.4)		
Yes	1614(56.7)	856(57.8)	758(55.6)		
BMI, n(%)				52.718	< 0.001^b^
Normal	1444(50.8)	837(56.5)	607(44.5)		
Underweight	139(4.9)	74(5.0)	65(4.8)		
Overweight	875(30.8)	414(28.0)	461(33.8)		
Obese	326(11.5)	125(8.4)	201(14.7)		
Missing, n(%)	61(2.1)	31(2.1)	30(2.2)		
Abdominal obesity, n(%)				209.466	< 0.001^c^
No	997(35.0)	703(47.5)	294(21.6)		
Yes	1848(65.0)	778(52.5)	1070(78.4)		
Health status, n(%)				18.363	< 0.001^b^
Good	728(25.6)	416(28.1)	312(22.9)		
Fair	1509(53.0)	786(53.1)	723(53.0)		
Poor	569(20.0)	257(17.4)	312(22.9)		
Missing, n(%)	39(1.4)	22(1.5)	17(1.2)		
Chronic disease, n(%)				6.163	0.046^b^
0	984(34.6)	532(35.9)	452(33.1)		
1	887(31.2)	474(32.0)	413(30.3)		
≥ 2	920(32.3)	449(30.3)	471(34.5)		
Missing, n(%)	54(1.9)	26(1.8)	28(2.1)		
Sleep duration (h/night),n(%)				32.984	< 0.001^b^
≥ 7and < 8	539(18.9)	294(19.9)	245(18.0)		
< 6	883(31.0)	401(27.1)	482(35.3)		
≥ 6and < 7	639(22.5)	382(25.8)	257(18.8)		
≥ 8and < 9	493(17.3)	268(18.1)	225(16.5)		
≥ 9	168(5.9)	87(5.9)	81(5.9)		
Missing, n(%)	123(4.3)	49(3.3)	74(5.4)		
Nap time(min/day), n(%)				10.475	< 0.001^b^
0	1204(43.3)	536(36.2)	668(49.0)		
> 0 and < 30	194(6.8)	104(7.0)	90(6.6)		
≥ 30 and < 60	265(9.3)	133(9.0)	132(9.7)		
≥ 60	1090(38.3)	661(44.6)	429(31.5)		
Missing, n(%)	92(3.2)	47(3.2)	45(3.3)		

Abbreviations: *SBP* Systolic blood pressure, *DBP* Diastolic blood pressure, *BMI* Body mass index

^a^*P*-values are derived from *t*-test

^b^*P*-values are derived from Wilcoxon rank sum test

^c^*P*-values are derived from Chi-square test

### Changes in the prehypertensive status of the sample

Table 2 shows the proportion of prehypertensive individuals who experienced a progression to hypertension or a return to normal BP. Approximately 38.8% of participants remained prehypertensive, 32.8% became normotensive, and 28.5% progressed to hypertensive. The rates of conversion of prehypertension to hypertension among men and women were 29.7% and 27.1%, respectively. There was a significant difference in age, systolic BP, education, abdominal obesity, alcohol habits, nap time, BMI, and the presence of chronic diseases among the three groups (*P* < 0.05).

**Table 2:** Changes in prehypertensive status of the full sample

Total(*n* = 2845)	Normal BP[n(%)] = 932 (32.8%)	pre-HT[n(%)] = 1103 (38.8%)	HT[n(%)] = 810 (28.5%)	*χ*^*2*^*/H/F*	*P* -value
SBP(mmHg)	127.35 ± 5.99	128.41 ± 5.86	129.42 ± 6.02	26.233	< 0.001^a^
DBP(mmHg)	75.75 ± 7.29	75.70 ± 7.09	75.96 ± 7.51	0.314	0.730^a^
Gender, n(%)				4.056	0.132^c^
Man	462(31.2)	579(39.1)	440(29.7)		
Women	470(34.5)	524(38.4)	370(27.1)		
Age group(years), n(%)				47.064	< 0.001^b^
45–54	353(38.2)	372(40.2)	200(21.6)		
55–64	368(32.5)	431(38.0)	335(29.5)		
65–74	158(27.9)	217(38.3)	192(33.9)		
≥ 75	52(24.0)	82(37.8)	83(38.2)		
Missing, n	1	1	0		
Marital status, n(%)				1.496	0.473^c^
Single	102(30.4)	130(38.7)	104(31.0)		
Married or cohabiting	830(33.1)	973(38.8)	706(28.1)		
Region, n(%)				3.167	0.205^c^
Urban	106(28.6)	153(41.4)	111(30.0)		
Rural	820(33.3)	946(38.4)	697(28.3)		
Missing, n	6	4	2		
Education level, n(%)				10.233	0.037^b^
Primary and lower	624(32.6)	716(37.4)	576(30.1)		
Junior/secondary/vocational school	280(33.3)	354(42.1)	206(24.5)		
College and higher	28(31.5)	33(37.1)	28(31.5)		
Current smoking status, n(%)				0.396	0.820^c^
Yes	344(32.1)	422(39.4)	306(28.5)		
No	588(33.2)	681(38.4)	504(28.4)		
Alcohol habits, n(%)				7.533	0.023^c^
Yes	411(30.8)	514(38.5)	411(30.8)		
No	519(34.5)	587(39.0)	399(26.5)		
Missing, n	2	2	0		
Social activity, n(%)				3.097	0.213^c^
No	404(32.8)	458(37.2)	369(30.0)		
Yes	528(32.7)	645(40.0)	441(27.3)		
BMI, n(%)				39.890	< 0.001^b^
Normal	519(35.9)	545(37.7)	380(26.3)		
Underweight	58(41.7)	49(35.3)	32(23.0)		
Overweight	264(30.2)	357(40.8)	254(29.0)		
Obese	68(20.9)	135(41.4)	123(37.7)		
Missing, n	23	17	21		
Abdominal obesity, n(%)				23.106	< 0.001^c^
No	378(37.9)	379(38.0)	240(24.1)		
Yes	554(30.0)	724(39.2)	570(30.8)		
Health status, n(%)				8.405	0.078^b^
Good	247(33.9)	292(40.1)	189(26.0)		
Fair	467(30.9)	601(39.8)	441(29.2)		
Poor	202(35.5)	198(34.8)	169(29.7)		
Missing, n	16	12	11		
Chronic disease, n(%)				18.262	< 0.001^b^
0	335(34.0)	408(41.5)	241(24.5)		
1	282(31.8)	352(39.7)	253(28.5)		
≥ 2	301(32.7)	318(34.6)	301(32.7)		
Missing, n	14	25	15		
Sleep duration (h/night), n(%)				6.182	0.627^b^
≥ 7and < 8	176(32.7)	218(40.4)	145(26.9)		
< 6	289(32.7)	334(37.8)	260(29.4)		
≥ 6and < 7	206(32.2)	260(40.7)	173(27.1)		
≥ 8and < 9	173(35.1)	178(36.1)	142(28.8)		
≥ 9	48(28.6)	74(44.0)	46(27.4)		
Missing, n	40	39	44		
Nap time(min/day), n(%)				9.084	0.011^b^
0	413(45.8)	486(45.3)	305(39.2)		
> 0 and < 30	61(6.8)	74(6.9)	59(7.6)		
≥ 30 and < 60	92(10.2)	95(8.9)	78(10.0)		
≥ 60	335(37.2)	418(39)	337(43.3)		
Missing, n	31	30	31		

Abbreviations: *BP* Blood pressure, *pre-HT* Prehypertension, *HT* Hypertension, *DBP* Diastolic blood pressure, *SBP* Systolic blood pressure, *BMI* Body mass index

^a^*P*-values are derived from One-way ANOVA test

^b^*P*-values are derived from Kruskal–Wallis test

^c^*P*-values are derived from Chi-square test

### Changes in prehypertensive status according to sex

Table 3 summarizes the proportions of the BP outcome statuses for prehypertensive men, 39.1% of whom remained prehypertensive, 31.2% of whom became normotensive, and 29.7% of whom progressed to hypertension. Systolic BP, age, marital status, current smoking status, BMI, abdominal obesity, health status, and chronic diseases were significantly associated with the development of hypertension (*P* < 0.05). Older age, being single, being a non-smoker, having worse health status, and having more chronic diseases were associated with higher rates of progression to hypertension (*P* < 0.05).

**Table 3:** Changes in prehypertensive status in men

Men(*n* = 1481)	Normal BP[n(%)] = 462 (31.2%)	pre-HT[n(%)] = 579 (39.1%)	HT[n(%)] = 440 (29.7%)	*χ*^*2*^*/H/F*	*P* -value
SBP(mmHg)	127.41 ± 5.84	128.42 ± 5.93	129.02 ± 5.91	8.623	< 0.001^a^
DBP(mmHg)	76.14 ± 7.22	76.09 ± 7.23	76.29 ± 7.27	0.098	0.907^a^
Age group(years), n(%)				32.640	< 0.001^b^
45–54	164(36.1)	187(41.2)	103(22.7)		
55–64	193(32.5)	226(38.0)	175(29.5)		
65–74	81(25.6)	126(39.9)	109(34.5)		
≥ 75	24(20.7)	39(33.6)	53(45.7)		
Missing, n	0	2	0		
Marital status, n(%)				14.768	< 0.001^c^
Single	26(19.4)	51(38.1)	57(42.5)		
Married or cohabiting	436(32.4)	528(39.2)	383(28.4)		
Region, n(%)				3.069	0.216^c^
Urban	49(26.2)	83(44.4)	55(29.4)		
Rural	408(31.7)	495(38.4)	385(29.9)		
Missing, n	5	1	0		
Education level, n(%)				8.950	0.062^b^
Primary and lower	252(29.4)	327(38.2)	277(32.4)		
Junior/secondary/vocational school	191(33.8)	231(40.9)	143(25.3)		
College and higher	19(31.7)	21(35.0)	20(33.3)		
Current smoking status, n(%)				6.464	0.039^c^
Yes	239(29.5)	340(42.0)	230(28.4)		
No	223(33.2)	239(35.6)	210(31.2)		
Alcohol habits, n(%)				5.202	0.074^c^
Yes	313(29.8)	410(39.0)	329(31.3)		
No	148(34.7)	168(39.3)	111(26.0)		
Missing, n	1	1	0		
Social activity, n(%)				1.697	0.428^c^
No	190(30.4)	238(38.1)	197(31.5)		
Yes	272(31.8)	341(39.8)	243(28.4)		
BMI, n(%)				27.976	< 0.001^b^
Normal	288(34.4)	308(36.8)	241(28.8)		
Underweight	28(37.8)	28(37.8)	18(24.3)		
Overweight	116(28.0)	181(43.7)	117(28.3)		
Obese	19(15.2)	52(41.6)	54(43.2)		
Missing, n	11	10	10		
Abdominal obesity, n(%)				15.743	< 0.001^c^
No	254(36.1)	261(37.1)	188(26.7)		
Yes	208(26.7)	318(40.9)	252(32.4)		
Health status, n(%)				11.358	0.023^b^
Good	143(34.4)	172(41.3)	101(24.3)		
Fair	232(29.5)	312(39.7)	242(30.8)		
Poor	80(31.1)	87(33.9)	90(35.0)		
Missing, n	7	8	7		
Chronic disease, n(%)				18.746	< 0.001^b^
0	175(32.9)	232(43.6)	125(23.5)		
1	138(29.1)	186(39.2)	150(31.6)		
≥ 2	142(31.6)	151(33.6)	156(34.7)		
Missing, n	7	10	9		
Sleep duration (h/night), n(%)				4.154	0.843^b^
≥ 7and < 8	97(33.0)	112(38.1)	85(28.9)		
< 6	120(29.9)	161(40.1)	120(29.9)		
≥ 6and < 7	116(30.4)	156(40.8)	110(28.8)		
≥ 8and < 9	94(35.1)	95(35.4)	79(29.5)		
≥ 9	24(27.6)	38(43.7)	25(28.7)		
Missing, n	11	17	21		
Nap time(min/day), n(%)				3.408	0.182^b^
0	179(39.8)	212(37.7)	145(34.4)		
> 0 and < 30	32(7.1)	37(6.6)	35(8.3)		
≥ 30 and < 60	49(10.9)	46(8.2)	38(9.0)		
≥ 60	190(42.2)	267(47.5)	204(48.3)		
Missing, n	12	17	18		

Abbreviations: *BP* Blood pressure, *pre-HT* Prehypertension, *HT* Hypertension, *SBP* Systolic blood pressure, *DBP* Diastolic blood pressure, *BMI* Body mass index

^a^*P*-values are derived from One-way ANOVA test

^b^*P*-values are derived from Kruskal–Wallis test

^c^*P*-values are derived from Chi-square test

Table 4 shows the proportion of the BP outcome statuses for prehypertensive women, 38.4% of whom remained prehypertensive, 34.5% of whom became normotensive, and 27.1% progressed to hypertension. Systolic BP, age, current smoking status, BMI, abdominal obesity, and nap time were significantly associated with the development of hypertension (*P* < 0.05). A higher proportion of women with baseline prehypertension who smoked or had excessive waist circumference progressed to having hypertension. (*P* < 0.05).

**Table 4:** Changes in prehypertensive status in women

Women(*n* = 1364)	Normal BP[n(%)] = 470 (34.5%)	pre-HT[n(%)] = 524 (38.4%)	HT[n(%)] = 370 (27.1%)	*χ2/H/F*	*P -*value
SBP(mmHg)	127.29 ± 6.14	128.39 ± 5.79	129.89 ± 6.11	19.424	< 0.001^a^
DBP(mmHg)	75.36 ± 7.36	75.28 ± 6.92	75.57 ± 7.78	0.178	0.837^a^
Age group(years), n(%)				20.472	0.002^b^
45–54	189(40.1)	185(39.3)	97(20.6)		
55–64	175(32.4)	205(38.0)	160(29.6)		
65–74	77(30.7)	91(36.3)	83(33.1)		
≥ 75	28(27.7)	43(42.6)	30(29.7)		
Missing, n	1	0	0	2.021	0.364^c^
Single	76(37.6)	79(39.1)	47(23.3)		
Married or cohabiting	394(33.9)	445(38.3)	323(27.8)		
Region, n(%)				1.661	0.436^c^
Urban	57(31.1)	70(38.3)	56(30.6)		
Rural	412(35.1)	451(38.4)	312(26.6)		
Missing, n	1	3	2		
Education level, n(%)				6.567	0.161^b^
Primary and lower	372(35.1)	389(36.7)	299(28.2)		
Junior/secondary/vocational school	89(32.4)	123(44.7)	63(22.9)		
College and higher	9(31.0)	12(41.4)	8(27.6)		
Current smoking status, n(%)				7.645	0.022^c^
Yes	105(39.9)	82(31.2)	76(28.9)		
No	365(33.2)	442(40.1)	294(26.7)		
Alcohol habits, n(%)				0.682	0.711^c^
Yes	98(34.5)	104(36.6)	82(28.9)		
No	371(34.4)	419(38.9)	288(26.7)		
Missing, n	1	1	0		
Social activity, n(%)				2.134	0.344^c^
No	214(35.3)	220(36.3)	172(28.4)		
Yes	256(33.8)	304(40.1)	198(26.1)		
BMI, n(%)				22.435	< 0.001^b^
Normal	231(38.1)	237(39.0)	139(22.9)		
Underweight	30(46.2)	21(32.3)	14(21.5)		
Overweight	148(32.1)	176(38.2)	137(29.7)		
Obese	49(24.4)	83(41.3)	69(34.3)		
Missing, n	12	7	11		
Abdominal obesity, n(%)				19.079	< 0.001^c^
No	124(42.2)	118(40.1)	52(17.7)		
Yes	346(32.3)	406(37.9)	318(29.7)		
Health status, n(%)				4.509	0.341^b^
Good	104(33.3)	120(38.5)	88(28.2)		
Fair	235(32.5)	289(40)	199(27.5)		
Poor	122(39.1)	111(35.6)	79(25.3)		
Missing, n	9	4	4		
Chronic disease, n(%)				4.997	0.288^b^
0	160(35.4)	176(38.9)	116(25.7)		
1	144(34.9)	166(40.2)	103(24.9)		
≥ 2	159(33.8)	167(35.5)	145(30.8)		
Missing, n	7	15	6		
Sleep duration (h/night), n(%)				6.363	0.607^b^
≥ 7and < 8	79(32.2)	106(43.3)	60(24.5)		
< 6	169(35.1)	173(35.9)	140(29.0)		
≥ 6and < 7	90(35.0)	104(40.5)	63(24.5)		
≥ 8and < 9	79(35.1)	83(36.9)	63(28.0)		
≥ 9	24(29.6)	36(44.4)	21(25.9)		
Missing, n	29	22	23		
Nap time(min/day), n(%)				7.661	0.022^b^
0	234(51.9)	274(53.6)	160(44.8)		
> 0 and < 30	29(6.4)	37(7.2)	24(6.7)		
≥ 30 and < 60	43(9.5)	49(9.6)	40(11.2)		
≥ 60	145(32.2)	151(29.5)	133(37.3)		
Missing, n	19	13	13		

Abbreviations: *BP* Blood pressure, *pre-HT* Prehypertension, *HT* Hypertension, *SBP* Systolic blood pressure, *DBP* Diastolic blood pressure, *BMI* Body mass index

^a^*P*-values are derived from One-way ANOVA test

^b^*P*-values are derived from Kruskal–Wallis test

^c^*P*-values are derived from Chi-square test

### Factors influencing the progression from prehypertension to hypertension

Among men, after adjusting for various covariates, the results of the multiple logistic regression analyses revealed that, compared with 45–54 years old respondents, those of older age (55–64 years: aOR = 1.414, 95%CI: 1.032–1.938; 65–74 years: aOR = 1.633, 95%CI: 1.132–2.355; ≥ 75 years: aOR = 2.974, 95%CI: 1.748–5.060) had a higher risk of developing hypertension. A significant negative association was observed between marital status (married or cohabitating) and the conversion from prehypertension to hypertension (aOR = 0.642, 95% CI: 0.418–0.985), and there was an association between obesity and progression to hypertension (aOR = 1.634, 95%CI: 1.022–2.611). One (aOR = 1.366, 95%CI: 1.004–1.859) or more than two (aOR = 1.568, 95%CI: 1.134–2.169) chronic diseases were risk factors for the progression from prehypertension to hypertension (Table 5).

**Table 5:** Adjusted odds ratios and 95% confidence intervals for the risk factors of progression from prehypertension to hypertension among men

Characteristics	*β*	*S.E*	*P-*value	a*OR*(95% *CI*)
Age group				
45–54	Ref			
55–64	0.347	0.161	**0.031**	1.414(1.032–1.938)
65–74	0.490	0.187	**0.009**	1.633(1.132–2.355)
≥ 75	1.090	0.271	** < 0.001**	2.974(1.748–5.060)
Marital status				
Single	Ref			
Married or cohabiting	-0.444	0.219	**0.042**	0.642(0.418–0.985)
Region				
Urban	Ref			
Rural	-0.070	0.196	0.721	0.932(0.634–1.370)
Education				
Primary and lower	Ref			
Junior/secondary/vocational school	-0.192	0.141	0.173	0.825(0.625–1.088)
College and higher	-0.130	0.420	0.756	0.878(0.385–2.001)
Alcohol habits				
No	Ref			
Yes	-0.173	0.143	0.225	0.841(0.636–1.112)
Current smoking				
No	Ref			
Yes	0.076	0.127	0.548	1.079(0.842–1.384)
Social activity				
No	Ref			
Yes	-0.102	0.131	0.437	0.903(0.698–1.168)
BMI				
Normal	Ref			
Underweight	-0.300	0.322	0.351	0.741(0.394–1.391)
Overweight	-0.043	0.170	0.800	0.958(0.687–1.336)
Obese	0.491	0.239	**0.040**	1.634(1.022–2.611)
Abdominal obesity				
No	Ref			
Yes	0.243	0.161	0.130	1.276(0.931–1.747)
Health status				
Good	Ref			
Fair	0.181	0.155	0.240	1.199(0.886–1.623)
Poor	0.239	0.213	0.262	1.271(0.836–1.93)
Chronic disease				
0	Ref			
1	0.312	0.157	**0.047**	1.366(1.004–1.859)
≥ 2	0.450	0.165	**0.007**	1.568(1.134–2.169)
Sleep duration				
≥ 7and < 8	Ref			
< 6	-0.044	0.185	0.811	0.957(0.666–1.374)
≥ 6and < 7	-0.110	0.184	0.549	0.896(0.625–1.284)
≥ 8and < 9	-0.017	0.199	0.931	0.983(0.665–1.452)
≥ 9	-0.181	0.302	0.548	0.834(0.461–1.508)
Nap time				
0	Ref			
0 > and < 30	0.238	0.251	0.343	1.269(0.776–2.074)
≥ 30 and < 60	0.119	0.232	0.608	1.126(0.715–1.774)
≥ 60	0.077	0.143	0.591	1.080(0.816–1.428)

Abbreviations: *β* Coefficient, *SE* Standard error; *BMI* Body mass index, *aOR* Adjusted odds ratio, *CI* Confidence interval, *Ref* reference

Among women, compared with 45–54 years old respondents, those of an older age (55–64 years: aOR = 1.755, 95%CI: 1.256–2.450; 65–74 years: aOR = 2.430, 95%CI: 1.605–3.678; ≥ 75 years: aOR = 2.037, 95% CI: 1.038–3.995) had a higher risk of developing hypertension. Being married or cohabiting was associated with the progression to hypertension (aOR = 1.662, 95%CI: 1.052–2.626), as was obesity (aOR = 1.874, 95%CI: 1.229–2.857). Napping for ≥ 30 and < 60 min (aOR = 1.682, 95%CI: 1.072–2.637) or ≥ 60 min (aOR = 1.387, 95%CI: 1.019-–1.889) were risk factors for the progression from prehypertension to hypertension (Table 6).

**Table 6:** Adjusted odds ratios and 95% confidence intervals for the risk factors of progression from prehypertension to hypertension among women

Characteristics	*β*	*S.E*	*P-*value	a*OR*(95% *CI*)
Age group				
45–54	Ref			
55–64	0.562	0.170	** < 0.001**	1.755(1.256–2.450)
65–74	0.888	0.212	** < 0.001**	2.430(1.605–3.678)
≥ 75	0.711	0.344	**0.039**	2.037(1.038–3.995)
Marital status				
Single	Ref			
Married or cohabiting	0.508	0.234	**0.030**	1.662(1.052–2.626)
Region				
Urban	Ref			
Rural	0.003	0.214	0.989	1.003(0.659–1.526)
Education				
Primary and lower	Ref			
Junior/secondary/vocational school	-0.183	0.188	0.330	0.832(0.575–1.204)
College and higher	0.591	1.197	0.621	1.806(0.173–18.871)
Alcohol habits				
No	Ref			
Yes	-0.069	0.168	0.679	0.933(0.672–1.296)
Current smoking				
No	Ref			
Yes	-0.095	0.177	0.593	0.910(0.643–1.287)
Social activity				
No	Ref			
Yes	-0.158	0.143	0.269	0.854(0.645–1.130)
BMI				
Normal	Ref			
Underweight	-0.022	0.396	0.955	0.978(0.45–2.125)
Overweight	0.333	0.173	0.054	1.395(0.995–1.958)
Obese	0.628	0.215	**0.003**	1.874(1.229–2.857)
Abdominal obesity				
No	Ref			
Yes	0.327	0.216	0.130	1.387(0.908–2.118)
Health status				
Good	Ref			
Fair	-0.041	0.175	0.813	0.959(0.681–1.352)
Poor	-0.134	0.231	0.561	0.875(0.556–1.374)
Chronic disease				
0	Ref			
1	-0.051	0.181	0.777	0.950(0.667–1.354)
≥ 2	0.319	0.181	0.078	1.376(0.964–1.963)
Sleep duration				
≥ 7and < 8	Ref			
< 6	0.32	0.202	0.114	1.377(0.926–2.047)
≥ 6and < 7	-0.016	0.228	0.944	0.984(0.629–1.539)
≥ 8and < 9	0.209	0.227	0.358	1.232(0.790–1.923)
≥ 9	0.095	0.327	0.771	1.100(0.579–2.089)
Nap time				
0	Ref			
0 > and < 30	0.255	0.289	0.377	1.291(0.732–2.275)
≥ 30 and < 60	0.52	0.23	**0.024**	1.682(1.072–2.637)
≥ 60	0.327	0.158	**0.038**	1.387(1.019–1.889)

Abbreviations: *β* Coefficient, *SE* Standard error, *BMI* Body mass index, *aOR* Adjusted odds ratio, *CI* Confidence interval, *Ref* reference

## Discussion

In this study, among the 2,845 eligible participants with prehypertension at baseline, 28.5% (440 men and 370 women) experienced progression to hypertension over the 2-year period. The conversion rate in a Strong Heart Study (SHS) cohort was 38% in prehypertensive participants over a 4-year follow-up period [28]. Sun found that after 10 years of monitoring the BP levels of people aged 35–64 years in Beijing, 52.6% experienced a conversion from a prehypertensive state to hypertension [29]. However, one prospective study conducted over 11.8 years found that 26.1% of prehypertensive patients developed hypertension [9]. Compared with the findings of other scholars, the proportion of individuals exhibiting a conversion from prehypertension to hypertension in the present study was relatively low. One possibility for this discrepancy may be that the follow-up periods of other follow-up studies examining the risk factors for predicting hypertension were relatively long [9, 30, 31], whereas in this study, the period was only 2 years. Therefore, it is possible that significant elevations of systolic and diastolic BPs were observed only in some study subjects. Despite this relatively short follow-up period, the present results still demonstrated that some prehypertensive subjects developed hypertension rapidly.

Similar to the findings of previous studies [30, 32, 33], the results revealed that older age and obesity were independent risk factors for the progression to hypertension among both men and women. With increasing age, the conversion rate to hypertension also increased. Several mechanisms are common to biologic aging and hypertension development, including inflammation, oxidative stress, and endothelial dysfunction [34]. Epigenetic age acceleration is significantly associated with cardiovascular diseases [35]. In the present study, men ≥ 75 years of age had a 2.953 times higher risk of conversion to hypertension; in women, the risk was 2.053 times higher. In the 65–74-year-old age group, the risk of progressing to hypertension was much higher in women than in men (2.444 vs. 1.631, respectively). A previous study demonstrated that BP starts to rise at about 30 years of age, and the prevalence of hypertension will reach up to 60–70% in the seventh decade of men’s life [36]. In contrast, among women, the prevalence of BP rises much more slowly with age before their menopause [37]. Subsequently, with the increasing pressure by the age of 65 or 70 years, more women may suffer from hypertension with ageing [36–38]. It appears that this may be a result of the loss of estrogen during the menopause period, leading to an increase in sympathetic nerve activity and adrenergic vasoconstrictor responsiveness [36, 37]. These factors are likely to contribute to a higher prevalence of hypertension among postmenopausal women.

This study found that obesity was a risk factor for progression from prehypertension to hypertension, a finding that is consistent with those of previous studies [36, 39]. Hypertension is closely linked to the prevalence, pathophysiology, and morbidity of obesity. Increased sympathetic nervous system activity has been shown to contribute to hypertension associated with obesity [39]. In recent years, the prevalence of obesity has continued to climb, particularly in developing countries. As obesity the single most important modifiable risk factor for prehypertension and hypertension [40], interventional studies must be carried out to investigate whether reducing obesity is beneficial in preventing the progression from prehypertension to hypertension in both women and men.

The present study revealed that having chronic disease was a risk factor for progressing from prehypertension to hypertension only among men. Previous studies have shown that some common chronic diseases (such as dyslipidemia and diabetes) are risk factors for hypertension and the progression from prehypertension to hypertension [31, 41, 42]. Therefore, individuals with more chronic diseases have an increased risk of this progression. Conversely, there was no significant association between chronic disease and the progression to hypertension among women. One possible reason for this outcome may be that women pay more attention to their health than men do [43]. Previous studies revealed that women are prone to maintaining their healthy behaviors, whereas men are inclined to forgo medical care and continue to practice their unhealthy behaviors [44, 45]. Therefore, when women suffer from chronic diseases, they may focus more on behaviors to improve their health, such as controlling their diet and increasing exercise, mitigating the effect of chronic diseases on hypertension.

Partaking in longer nap times (≥ 30 min) was a significant risk factor for conversion to hypertension only among women in this study. Some studies have found a relationship between nap duration and objective measures of health status [46]. For example, a Dongfeng-Tongji cohort study reported that a longer nap duration (≥ 60 min) was associated with hypertension [5]. Nap may partially compensate for sleep loss among those who stay up late at night and counteract daytime sleepiness or fatigue which might be the early clinical manifestations of the underlying diseases [47]. Although excessive daytime sleepiness increases risk to somatic health, including BP dysregulation [48], longer nap times were not a risk factor for the progression from prehypertension to hypertension among men in the present study, although the reason for this finding requires further investigation.

Among women, married or cohabiting individuals had a higher risk for conversion to hypertension, which is consistent with the results of studies in Ghana and India [49, 50]. One social causation hypothesis argues that an individual’s marital status determines one’s exposure to their social and economic conditions that influence health outcomes [49, 51]. Previous research results show that domestic stressors have a greater effect on ambulatory BP in women than in men [52]. In traditional Chinese culture, many women not only have to work, but they also need to invest more energy into the family. Wives who take care of their families full-time rely on their husband’s economic and emotional support. Catecholamine level, which positively correlate with BP, remains high among working, married women, even after leaving their work environment. It indicates that home stressors may continue to influence their sympathetic activation at home. It is urgent to identify the possible pathophysiological mechanism [53]. Previous studies showed that marriage was a risk factor for hypertension in men [54, 55]; however, the present findings showed that marriage was a protective factor for the conversion of prehypertension to hypertension among men. The reason for this may be that men who were married could easily get support from their spouses. In traditional Chinese culture, men often seek a meaningful challenges in their workplace, whereas women are more likely to focus on their family, including family members’ health [27]. Therefore, once men are diagnosed with chronic diseases (such as prehypertension), they may receive more care from their spouses.

This study has several limitations. First, because some independent variables were based on self-reported responses, it was possible that there may have been response bias. Second, because some samples were excluded from the analysis because the participants took antihypertensive drugs or lost to follow-up because of death, the odds of becoming hypertensive might be underestimated [56]. Third, some samples were excluded from the analysis due to the missing BP data, which could affect our findings. Fourth, hypertension status was determined via BP calculations based on three readings collected in a single day, which might have compromised the results due to detection bias. Lastly, because the outcome is more than 10% in this manuscript, the estimated odds ratios through multiple logistic regression analysis may overestimate the relative risk [57].

### Policy implications

Based on our findings, we suggest that policy-makers and community medical staff should (1) strengthen hypertension monitoring to achieve early detection and intervention, and reduce the rate of progression from pre-hypertension to hypertension, especially for older adults; (2) pay more attention to BP status of men with chronic diseases or single; (3) actively promote the idea that men should share the household work to reduce the stress of caring for women; (4) strengthen community health education to avoid the longer naps (≥ 30 and < 60 min), especially for female adults; (5) develop community health education programs aimed to reduce the prevalence of obesity.

## Conclusions

Chinese middle-aged and elderly individuals experienced a risk of conversion from prehypertension to hypertension over a 2-year period. Older age and obesity were factors influencing the conversion of prehypertension to hypertension in both men and women. Among men, the number of chronic diseases was a risk factor, whereas being married or cohabiting was a protective factor for the progression from prehypertension to hypertension. Among women, being married or cohabiting and napping for ≥ 30 min were the factors influencing the progression to hypertension. Prehypertensive individuals should receive lifestyle interventions such as body weight control and frequent BP monitoring.

## Data Availability

The datasets analysed for the current study are available in the CHARLS (https://charls.charlsdata.com/pages/data/111/zh-cn.html).

## Abbreviations

aOR: Adjusted odds ratio
BMI: Body mass index
BP: Blood pressure
CHARLS: China health and retirement longitudinal study
CI: Confidence interval
DBP: Diastolic blood pressure
HT: Hypertension
pre-HT: Prehypertension
SBP: Systolic blood pressure
SD: Standard deviations
SE: Standard error
SPSS: Statistical package for the social sciences
